# Supplementary material for: Fermi-crossing Type-II Dirac fermions and topological surface states in NiTe2
Source: Sci Rep. 2020 Jul 31;10:12957. doi: 10.1038/s41598-020-69926-8 (PMC7395785; doi:10.1038/s41598-020-69926-8)
Supplement: Supplementary file 1 — Supplementary information. [file 41598_2020_69926_MOESM1_ESM.docx]

***Supplementary Information***

**Fermi-crossing Type-II Dirac fermions and topological surface states in NiTe_2_**

*Saumya Mukherjee^1, 2,*^, Sung Won Jung^1^, Sophie F. Weber^3,4^, Chunqiang Xu^5^, Dong Qian^6^ _,_ Xiaofeng Xu^5^, Pabitra K. Biswas^7^, Timur K. Kim^1^, Laurent C. Chapon^1^, Matthew D. Watson^1^, Jeffrey B. Neaton^3,4 ,8^, Cephise Cacho^1^*

^1^Diamond Light Source, Oxfordshire OX11 0DE, United Kingdom.

^2^Clarendon Laboratory, Department of Physics, University of Oxford, Parks Road, Oxford OX1 3PU, United Kingdom

^3^Department of Physics, University of California, Berkeley, California 94720, USA

^4^Molecular Foundry, Lawrence Berkeley National Laboratory, Berkeley, California 94720, USA

^5^ Department of Applied Physics, Zhejiang University of Technology, Hangzhou 310023, China

^6^School of Physics and Astronomy, Shanghai Jiao Tong University, Shanghai 200240, China

^7^ISIS facility, STFC Rutherford Appleton Laboratory, Harwell Science and Innovation Campus, Oxfordshire, OX11 0QX, United Kingdom

^8^Kavli Energy Nanosciences Institute at Berkeley, CA, 94720, USA

*Correspondence and requests for materials should be addressed to S.M. (email: Saumya.mukherjee@diamond.ac.uk)

In Supplementary information we present the calculated spin texture of TSS0 and TSS2 showing the spin components along *x*, *y* and *z* directions (Fig. S1). A comparative plot of ARPES experimental data with respect to DFT calculations for GGA+ *U* is added. It shows that the best agreement between experimental data and calculations is achieved at U = 0 (see Fig. S2).

Figures:


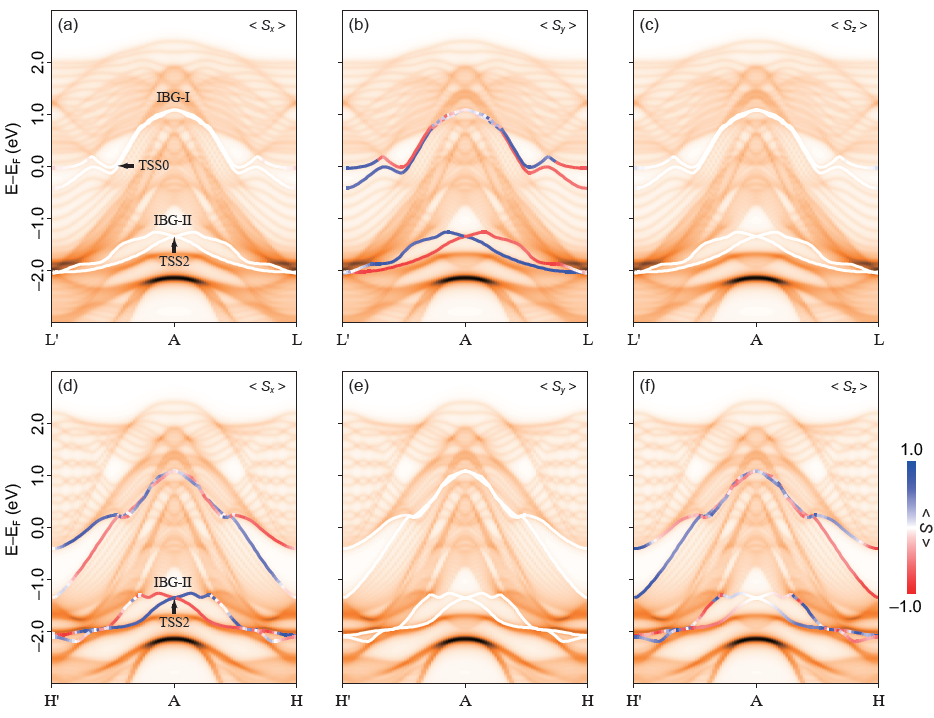


S1: Calculated spin texture of surface states showing spin components <*S_x_*> (a, d), <*S_y_*> (b, e) and <*S_z_*> (c, f).


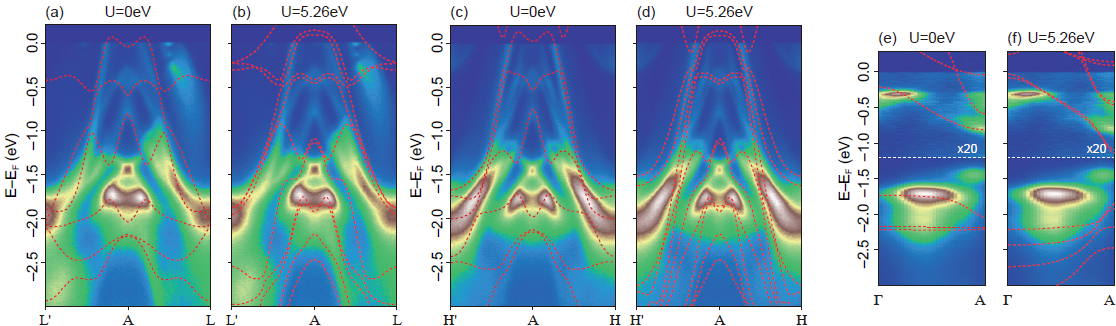


S2: Spectral band dispersion along in-plane $\text{L}\text{'}\text{-A-L}$ (a, b) and $\text{H}\text{'}\text{-A-H}$ (c, d) direction, probed with photon energy *h*ν = 99 eV, and along Γ-A direction (e, f). DFT band calculations with GGA + U = 0 eV (a, c, e) and U = 5.26 eV (b, d, f) are overlaid on the spectra as red dotted line. With finite U-parameter the agreement with experimental data do not improve and additional bands appear which are absent in the experimental data (b, d, f).
